# Brain magnetic resonance imaging and cognitive alterations after ablation in patients with atrial fibrillation

**DOI:** 10.1038/s41598-021-98484-w

**Published:** 2021-09-23

**Authors:** Natsuko Kato, Kanako Muraga, Yoshinori Hirata, Akihiro Shindo, Keita Matsuura, Yuichiro Ii, Mariko Shiga, Ken-ichi Tabei, Masayuki Satoh, Satoshi Fujita, Tomoyuki Fukuma, Yoshihiko Kagawa, Eitaro Fujii, Maki Umino, Masayuki Maeda, Hajime Sakuma, Masaaki Ito, Hidekazu Tomimoto

**Affiliations:** 1 Department of Neurology, Mie University Graduate School of Medicine, 2-174, Edobashi, Tsu, Mie Japan; 2 Department of Dementia Prevention and Therapeutics, Mie University Graduate School of Medicine, Tsu, Japan; 3 Department of Cardiology and Nephrology, Mie University Graduate School of Medicine, Tsu, Japan; 4 Department of Radiology, Mie University Graduate School of Medicine, Tsu, Japan; 5 Department of Neuroradiology, Mie University Graduate School of Medicine, Tsu, Japan; 6 Department of Neurologic Science, Graduate School of Medicine, Nippon Medical School, Tokyo, Japan

**Keywords:** Cardiology, Neurology, Risk factors

## Abstract

Catheter ablation is an important non-pharmacological intervention for atrial fibrillation (AF), but its effect on the incidence of asymptomatic cerebral emboli and long-term effects on cognitive function remain unknown. We prospectively enrolled 101 patients who underwent AF ablation. Brain magnetic resonance imaging (MRI) (72 patients) and neuropsychological assessments (66 patients) were performed 1–3 days (baseline) and 6 months after ablation. Immediately after ablation, diffusion-weighted MRI and 3-dimensional double inversion recovery (3D-DIR) detected embolic microinfarctions in 63 patients (87.5%) and 62 patients (86.1%), respectively. After 6 months, DIR lesions disappeared in 41 patients. Microbleeds (MBs) increased by 17%, and 65% of the de novo MBs were exactly at the same location as the microinfarctions. Average Mini-Mental State Examination scores improved from 27.9 ± 2.4 to 28.5 ± 1.7 (*p* = 0.037), and detailed neuropsychological assessment scores showed improvement in memory, constructional, and frontal lobe functions. Ejection fraction, left atrial volume index and brain natriuretic peptide level improved from baseline to 3–6 months after ablation. Despite incidental microemboli, cognitive function was preserved 6 months after ablation.

## Introduction

Dementia has remarkable clinical and social impacts worldwide^1^, while symptoms of dementia, such as memory deficits and executive dysfunction, interfere with activities of daily life. The two main causes of dementia are Alzheimer’s disease (AD) and vascular dementia (VaD). Both AD and VaD have been reported as the extremes of the same spectrum^2^, and vascular risk factors are closely associated not only with VaD but also AD^3^.

Atrial fibrillation (AF) contributes to the incidence and outcome of ischemic stroke^4^ as well as the occurrence of cognitive impairment/dementia^5–9^. These AF risks are increased in elderly individuals because the prevalence of AF increases linearly with age^10^. Several studies have reported that the risk of dementia is higher with AF but is downregulated by catheter ablation^11^ or administration of oral anticoagulant treatments for AF^12^.

AF is associated with an increased risk of dementia even in patients without a history of stroke, suggesting that dementia may be caused by other factors than stroke, such as subclinical cerebrovascular disease^13^. Several mechanisms have been proposed to explain the association between cognitive impairment and AF, including chronic cerebral hypoperfusion, microembolism, cerebral microbleeds (MBs), vascular inflammation, and brain atrophy^8,14^.

Cerebral MBs are mostly caused by small vessel disease, which refers to a group of conditions affecting the cerebral vessels, comprising the small arteries, arterioles, capillaries, and postcapillary venules, and is closely associated with dementia^2^. In patients with AF and small vessel disease, magnetic resonance imaging (MRI) of the brain shows various vascular lesions, such as cerebral MBs, white matter hyperintensities, and lacunar infarcts^15^. Moreover, AF is related to a decrease in brain volume^16^. Although these complex mechanisms may underlie the association between AF and dementia, no prospective studies to date have been conducted to determine the mechanism that leads to cognitive decline and dementia.

The mainstay of treatment in patients with AF includes prevention of ischemic stroke, administration of anti-arrhythmic medications, and non-pharmacological intervention. Pharmacological treatment with rate- and rhythm-control showed no significant differences in cognitive function over a 3-year follow-up period^17^. Catheter ablation is an important non-pharmacological intervention for AF. Although clinical data suggests that catheter ablation is superior to current pharmacological treatment, it is associated with procedural complications such as periprocedural transient ischemic attack (TIA) and cerebrovascular accidents^18^. Moreover, the incidence rates of asymptomatic cerebral emboli associated with catheter ablation for AF have been reported to range from 1.7 to 38% according to previous studies^19^.

The long-term effect of catheter ablation on cognitive function remains to be elucidated. Bunch et al.^11^ retrospectively reviewed the medical records of patients with AF who underwent catheter ablation. They found a significantly lower incidence and risk of dementia, including both VaD and AD, in AF patients treated with catheter ablation than in those without ablation^11^. On the contrary, a prospective study by Kochhauser et al.^20^ found no significant differences in the neuropsychological findings before and immediately after or 6 months after ablation. However, the effect of catheter ablation on microembolic infarctions was not evaluated in this study, thereby making it difficult to determine its net effect on cognitive function. In this prospective study, we evaluated whether catheter ablation has a protective effect on cognitive function by evaluating the incidence of cerebral microembolism as well as the neuropsychological outcome after catheter ablation in patients with AF.

## Methods

### Study protocol

This prospective study was approved by the ethical review board of the Mie University Hospital (permit number 3038), and all subjects provided written informed consent. All methods were performed in accordance with the Declaration of Helsinki. A total of 101 patients who were admitted to the hospital for AF ablation were recruited from the Department of Cardiology of Mie University Hospital between August 2018 and September 2019.

We obtained clinical, laboratory, and imaging data corresponding to baseline and follow-up timepoints after catheter ablation. Patient characteristics including age, sex, hypertension, status of diabetes mellitus and dyslipidemia, history of stroke and/or TIA, and AF type were recorded. Cardiac function was assessed by measuring ejection fraction (EF) and left atrial volume index (LAVI) using transthoracic echocardiography, and brain natriuretic peptide (BNP) levels before ablation (baseline) and during the 3 to 6-month period after ablation (follow-up). For the measurement of LAVI, we used a biplane (apical 2 chamber view and 4 chamber view) modified Simpson method.

### Ablation procedure

Catheter ablation was performed as described previously^21^. An electrophysiological study was performed in the post-absorptive state under light sedation. Trans-esophageal echocardiography was performed to exclude the possibility of LA appendage thrombus just before ablation in all patients. After internal jugular and femoral vein punctures, a heparin bolus (100 U/kg) was administered, and continuous heparin infusion provided thereafter to maintain an activated clotting time of 250–350 s. A diagnostic duodecapolar catheter was placed in the coronary sinus via the jugular vein. Three long sheaths were inserted through the femoral vein and introduced in the LA through a single transseptal puncture guided by intracardiac echocardiography. Eicosapolar circumferential catheter (Lasso 2515, Biosense Webster, Diamond Bar, CA, USA) and multi-spline mapping catheter (PentaRay, Biosense Webster, Diamond Bar, CA, USA) were introduced in the LA through the transseptal long sheaths. All imaging was performed using a biplane flat-panel detector angiographic suite (Allura Xper FD10/10 angio system; Philips Healthcare, Best, Netherlands). Electroanatomical mapping was performed using the CARTO3 mapping system (Biosense Webster, Diamond Bar, CA, USA). Radiofrequency ablation was performed with an irrigated catheter (EZ Steer Thermocool, Biosense Webster, Diamond Bar, CA, USA) using 0.9% normal saline and a point-by-point technique. Extensive encircling pulmonary vein isolation (EEPVI) was performed in patients with paroxysmal AF, and entrance and exit blocks documented in all cases using Lasso2515 and PentaRay multipolar catheters. In addition to EEPVI, patients with persistent AF received LA posterior wall isolation; additional linear ablation along the LA roof to connect the left superior pulmonary vein to the right superior pulmonary vein and linear ablation along the LA floor to connect the inferior margin of the left inferior pulmonary vein to the right inferior pulmonary vein were performed to gain a block into the posterior wall. Bidirectional block was confirmed across all linear ablations using differential pacing techniques. If common atrial flutter was induced by atrial burst or extra stimulus pacing, cavotricuspid isthmus line ablation was performed in patients with both paroxysmal AF and persistent AF.

### MRI protocol

The MRI studies were performed at 1–3 days (baseline) and 6 months after ablation (follow-up) with a 3 T MR unit (Ingenia, Philips Medical System, The Netherlands) using a 32-channel phased-array head coil. We used diffusion-weighted imaging (DWI), 3-dimentional (3D) fluid-attenuated inversion recovery (3D-FLAIR), 3D double inversion recovery (3D-DIR), and 3D T1-weighted imaging (3D-T1WI) to detect microemboli^22,23^. Acute microinfarctions were diagnosed with DWI sequences, while chronic microinfarctions were evaluated with 3D-DIR, 3D-FLAIR, and 3D-T1WI sequences. To detect MBs, susceptibility-weighted imaging (SWI) was used.

3D-DIR parameters were as follows: FOV, 250 mm; matrix, 208 × 163; thickness, 1.3 mm; TR (ms)/TE (ms), 5500/247; TI (ms), 2550/450; NEX, 2; and acquisition time, 5 min 13 s. 3D-FLAIR parameters were as follows: FOV, 250 mm; matrix, 256 × 184; thickness, 1.14 mm; TR (ms)/TE (ms), 6000/390; TI, 2000 ms; NEX, 2; and acquisition time, 4 min 42 s. 3D-T1W imaging used turbo-field echo sequence and the following parameters: FOV, 260 mm; matrix, 288 × 288; thickness, 0.9 mm; TR (ms)/TE (ms), 8.4/4.7; NEX, 1; FA, 10°; and acquisition time, 4 min 56 s. SWI parameters were as follows: FOV, 230 mm; matrix, 384 × 300; thickness, 2 mm; TR (ms)/TE (ms), 31/7.2; NEX, 1; FA, 17°; and acquisition time, 4 min 52 s. And DWI parameters: FOV, 220 mm; matrix, 112 × 168; thickness, 3 mm without gap; TR (ms)/TE (ms), 5800/87; b value = 1,000 s/mm^2^; NEX, 1; three orthogonal diffusion directions; and acquisition time, 1 min 10 s.

All MR images were independently analyzed by two radiologists blinded to the patients’ clinical status.

### Management of oral anticoagulants

All patients were taking direct oral anticoagulants (DOACs) or warfarin before ablation. Warfarin was continued during the ablation procedure, and DOACs were omitted only on the day of ablation and continued until 3 months after ablation in all patients. If there was no AF recurrence > 3 months after ablation, the CHADS2 score was calculated. If the CHADS2 score was 0 or 1, oral anticoagulants were stopped. If the CHADS2 score was 2 to 6, oral anticoagulants were continued because of the higher risk of cerebral infarction in these patients.

### Neuropsychological assessment

To quantify intellectual function, the Mini-Mental State Examination (MMSE) and the Japanese Raven’s colored progressive matrices (RCPM) were administered. RCPM measures not only intelligence but also patients’ performance time, which reflects psychomotor speed. Memory was evaluated using the Rivermead Behavioral Memory Test (RBMT), which consisted of immediate and delayed recall of a short story. The assessment of constructional ability was based on the method described by Strub and Black^24^. A cube was shown to the examinees, and they were asked to draw it. Their drawing was scored by assigning one of 4 possible grades (0: poor, 1: fair, 2: good, and 3: excellent). Mie Constructional Apraxia Scale (MCAS) was also used for the assessment of constructional visuospatial ability^25^. The MCAS was invented to assess constructional disabilities by checking not only the shape of a drawn Necker cube but also the drawing process, with larger scores corresponding to more severe symptoms. Frontal function was assessed on the basis of two types of tasks: word fluency (WF) and trail-making test A,B (TMT-A,B). The WF test consisted of two domains: category and letters. For the categorical WF test, subjects were asked to name as many animals/vegetables as possible in one minute. For the letter WF test, subjects were asked to name objects that begin with each of 4 phonemes *ka*, *sa*, *ta*, and *te*. We used the average scores of these 4 phonemes for statistical analysis. It is generally accepted that the cognitive processing of categorical and letter WF is somewhat different; categorical WF is more reflective of memory function than letter WF^26^. TMT is a test of visual scanning speed with two parts. Part A consists of 25 circles numbered from 1 to 25 distributed on a piece of paper. The task is to “connect the dots” as quickly as possible. Part B consists of 25 circles with the numbers 1 to 13 and letters in sequence. The score corresponds to the number of seconds required to finish each part.

### Statistical analysis

For the analysis of differences in demographic characteristics, the Mann–Whitney U test and the χ-squared test were used. Differences in neuropsychological scores and cardiac function between the baseline and follow-up timepoints were analyzed using paired t-test or Mann–Whitney U test, and the improvement in MMSE score according to the type of AF and past history was valuated using Mann–Whitney U test. To test the correlations of neuropsychological test score changes with age, EF, LAVI, and BNP, Pearson’s and Spearman’s correlation coefficient was used. Differences showing a *p* value < 0.05 were considered statistically significant. Statistical analyses were performed using IBM SPSS Statistics software version 24 (IBM Corp., Armonk, NY, United States) (“https://www.ibm.com/products/spss-statistics”).

## Results

### Patient characteristics

**Figure 1:**
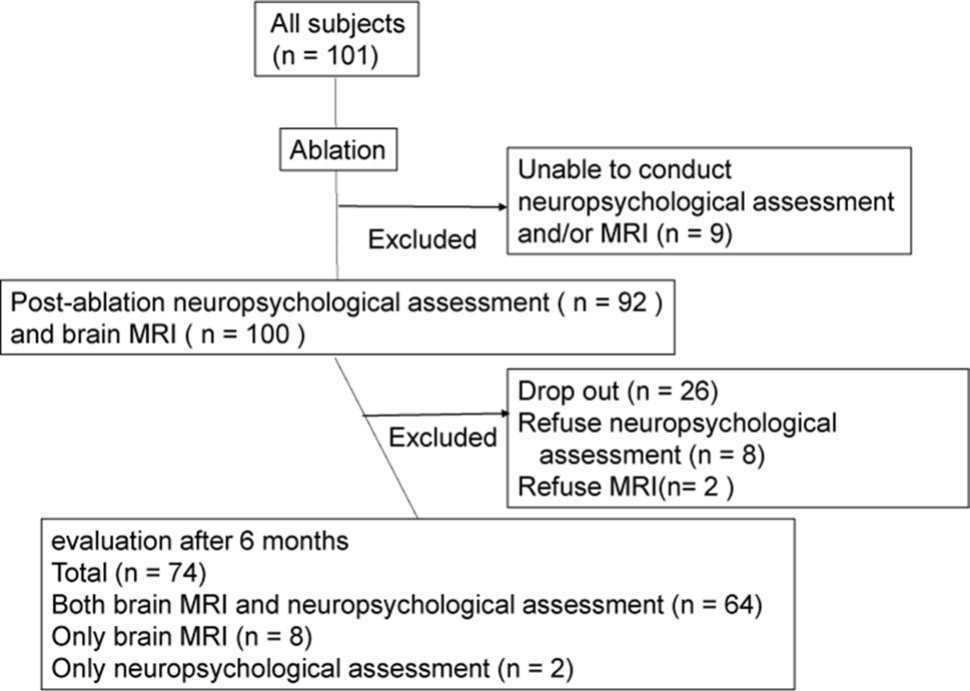
Study protocol. We recruited 101 patients; 9 were excluded because they were unable to conduct neuropsychological assessment and/or brain MRI. Therefore, one hundred patients underwent MRI and 92 underwent neuropsychological assessment at baseline. At 6 months after ablation, 27 patients dropped out, 2 declined to undergo MRI, and 8 declined to perform neuropsychological assessment. Finally, 72 and 66 patients underwent MRI and neuropsychological assessment, respectively. *MRI* Magnetic resonance imaging.

None of the patients showed neurological deficits immediately after ablation. At baseline, 100 patients underwent MRI and 92 underwent neuropsychological assessments. One patient could not undergo MRI because he previously underwent coil embolization for a cerebral arteriovenous malformation. Nine patients were unable to undergo neuropsychological assessment because of lack of time for assessment before discharge. At 6 months after ablation, 72 patients underwent MRI and 66 underwent neuropsychological assessment. Twenty-seven patients dropped out, 2 declined to undergo MRI, and 8 declined the neuropsychological assessment (Fig. 1). We analyzed the data of a total of 74 patients who underwent MRI and/or neuropsychological assessment both at baseline and 6 months after ablation. Sixty-four patients underwent both MRI and neuropsychological assessment. Eight patients underwent only MRI because they did not have sufficient time to finish the neuropsychological assessment after ablation. Two patients experienced claustrophobia while undergoing MRI at baseline and underwent only neuropsychological assessment after 6 months.

Cardiac function was evaluated in all patients before ablation, and repeated in 40 patients at 3–6 months after ablation. Thirty-two out of 72 patients were lost to follow-up because they were discharged to the care of their primary physicians at 1–2 months after ablation.

Patients’ baseline characteristics are shown in Table 1. The mean age at baseline was 68.3 ± 10.0 (range 32–86), and 53 of the patients were men (71.6%). Forty-one patients (55.4%) had non-paroxysmal AF, and 33 paroxysmal AF. All patients were using oral anticoagulants (DOAC:warfarin = 68:6) before ablation, and 52 patients (70.3%) continued oral anticoagulant use even at 6 months after ablation. Nine patients (12.2%) had AF recurrence within 6 months after ablation, and all had been taking oral anticoagulants at the time of AF recurrence. Thirty-eight patients had hypertension (51.3%), 10 had diabetes mellitus (13.5%), 22 had dyslipidemia (29.7%), and 5 had a history of stroke or TIA (6.7%). None had any neurological abnormalities after ablation.

**Table 1:** Demographic features of the patients.

	Persistent AF	Paroxysmal AF	Total	*p*
Sample size, n	41 (55.4%)	33 (44.6%)	74	
Mean age, y	68.4 ± 9.5 [41–86]	68.1 ± 9.5 [32–84]	68.3 ± 10.0 [32–86]	0.935
Male, n (%)	33 (80.5)	20 (60.6)	53 (71.6)	0.052
History of ablation, n (%)	10 (24.4)	9 (23.3)	19 (25.6)	0.492
Recurrence of AF within 6 months after ablation (%)	5 (12.2)	4 (12.1)	9 (12.2)	0.639
Use of anticoagulant at 6 months after ablation (%)	34 (82.9)	18 (54.5)	52 (70.3)	0.011*
Hypertension, n (%)	22 (53.7)	16 (48.5)	38 (51.3)	0.417
Diabetes mellitus, n (%)	8 (19.5)	2 (6.1)	10 (13.5)	0.088
Dyslipidemia, n (%)	11 (26.8)	11 (33.3)	22 (29.7)	0.361
History of stroke/TIA, n (%)	1 (2.4)	4 (12.1)	5 (6.7)	0.119

*AF* Atrial fibrillation, *TIA* Transient ischemic attack.

### Brain MRI findings after ablation

**Figure 2:**
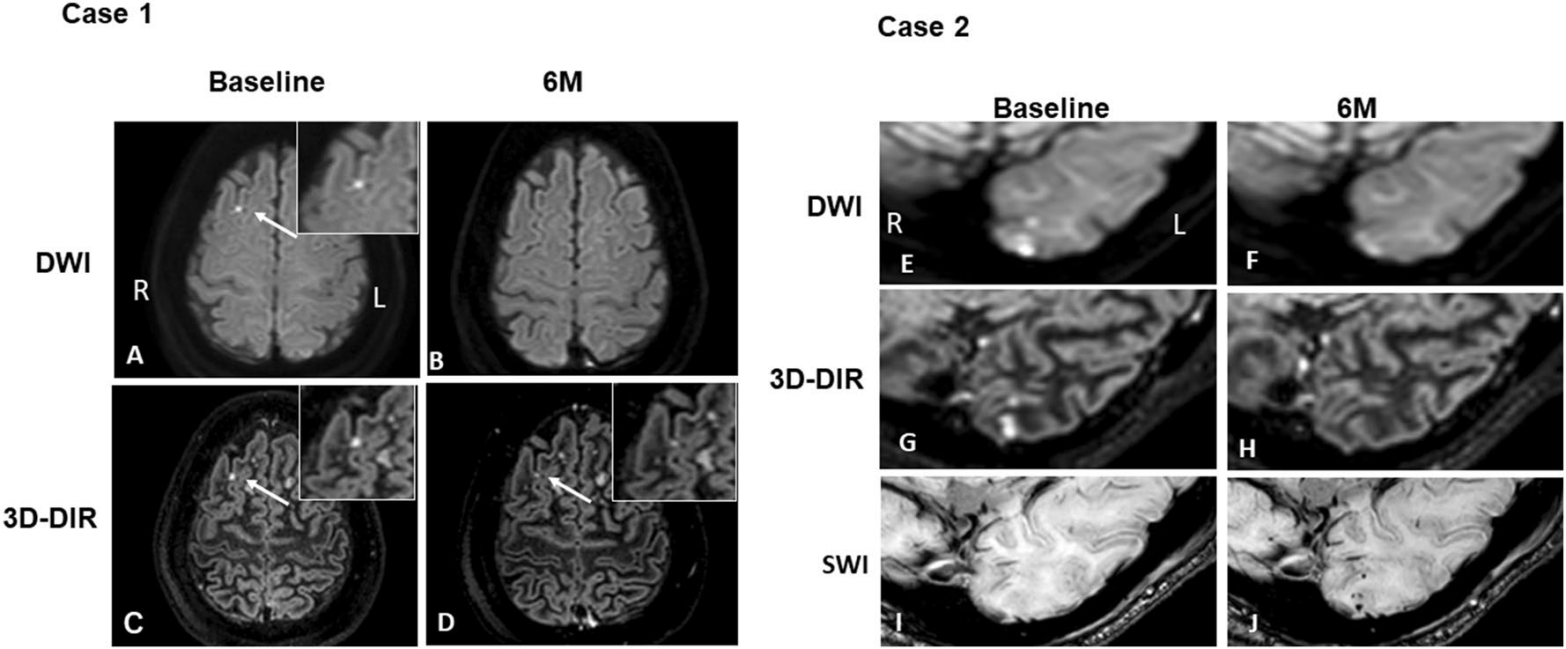
Brain MR images. Case 1: Brain MR images of a female patient. DWI (**A**, **B**), 3D-DIR findings at baseline (**C**) and after 6 months (**D**). An embolic infarct was detected by DWI and 3D-DIR after ablation. In follow-up MRI, the lesion was still detected by 3D-DIR. Case 2: Brain MR images of a male patient. DWI (**E**, **F**), 3D-DIR (**G**, **H**), SWI (**I**, **J**) findings at baseline (left) and after 6 months (right). The embolic microinfarctions detected by DWI and 3D-DIR after ablation disappeared at follow-up, but SWI detected de novo microbleeds exactly at the same location where the infarcts were detected at baseline. *DWI* Diffusion-weighted imaging, *3D-DIR* 3-Dimensional double inversion recovery, *SWI* Susceptibility-weighted imaging.

Figure 2 shows two representative patterns of MRI findings. Case 1 is a female patient who had an embolic microinfarct detected in the right frontal cortex by DWI and 3D-DIR after ablation. After 6 months, the lesion persisted on 3D-DIR. No signal changes suggesting hemorrhage were observed on SWI. Case 2 is a male patient who had embolic microinfarctions detected by DWI and 3D-DIR after ablation. The microinfarctions disappeared after 6 months, but SWI detected de novo MBs exactly at the same location where the infarct was detected at baseline.

**Figure 3:**
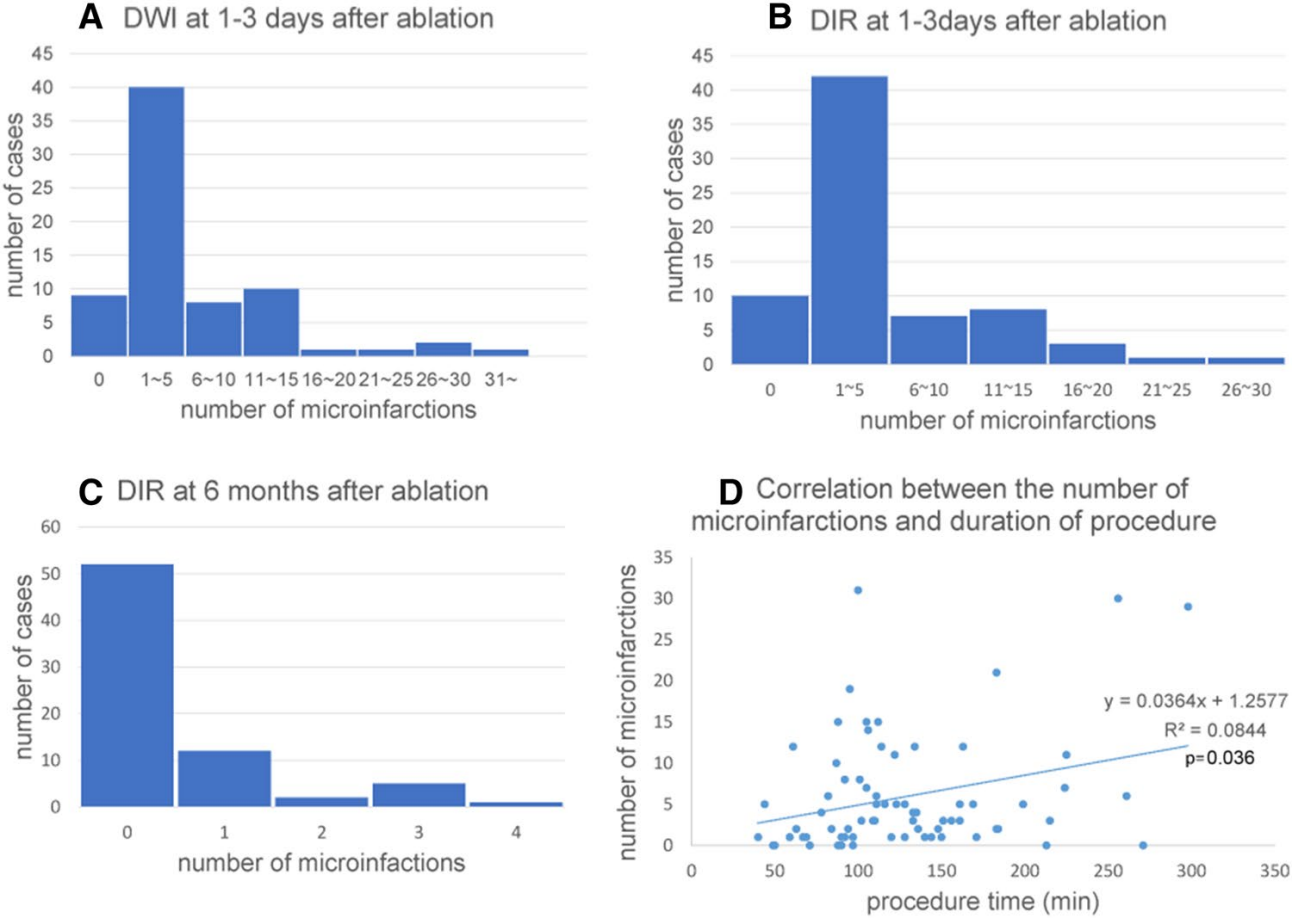
Number of DWI (**A**) and DIR (**B**) positive lesions per case at baseline, and DIR positive lesions per case at 6 months after ablation (**C**). At baseline, one to five lesions were most frequent, however, most lesions disappear on follow-up DIR images. A mild positive correlation was observed between the number of microinfarctions and duration of ablation procedure (*p* = 0.036) (**D**).

At baseline, DWI detected embolic microinfarctions in 63 out of 72 patients (87.5%), and 3D-DIR detected embolic lesions in 62 patients (86.1%). After 6 months, the embolic microinfarctions detected by 3D-DIR disappeared in 41 out of 62 patients (66.1%). Figure 3A–C shows the number of DWI/DIR positive lesions per case at baseline and DIR positive lesions per case at 6 months after ablation. At baseline, one to five lesions were most frequent. The median number of lesions was 3.0 (interquartile range, 6.25). Most lesions disappeared on follow-up DIR images.

At baseline, DWI detected a total of 421 embolic microinfarctions (cortical: subcortical and deep = 361: 60), and 3D-DIR detected 362 (cortical: subcortical and deep = 307: 55). Most lesions in cortical regions frequently showed a diameter < 5 mm, which infrequently reached 10 mm. Follow-up DIR images after 6 months detected 35 emboli (cortical: subcortical and deep = 18: 17). The residual rate was 5.8% (18 of 307) in the cortical region, and 30.9% (17 of 55) in subcortical regions (Table 2).

**Table 2:** Comparison between findings at baseline and 6 months after ablation.

		Baseline	6 M	*p*
**MRI findings**				
DWI positive patients, n (%)		63 (87.5)	0 (0)	Not applicable
DWI positive lesions, n		421	0	
Cortical		361	0	
Subcortical, deep		60	0	
DIR positive patients, n (%)		62 (86.1)	21 (29.2)	
DIR positive lesions, n		362	35	
Cortical		307	18	
Subcortical, deep		55	17	

Neuropsychological test	Normal range	Baseline	6 M	*p*
MMSE	≧ 24	27.9 ± 2.39	28.5 ± 1.98	0.037*
RCPM	Age dependent	31.4 ± 4.0	31.8 ± 3.1	0.503
RCPM time (s)	Age dependent	391 ± 92.2	392 ± 116	0.69
RBMT immediate recall	Age dependent	10.1 ± 3.4	14.3 ± 4.2	< 0.001*
RBMT delayed recall	Age dependent	7.8 ± 2.98	13.1 ± 4.0	< 0.001*
Necker’s cube		2.3 ± 0.5	2.4 ± 0.6	0.157
MCAS	≤ 2	2.4 ± 1.4	1.8 ± 1.3	< 0.001*
TMT-A	Age dependent	105 ± 31.2	97.8 ± 37.1	0.09
TMT-B	Age dependent	160.2 ± 146	141.1 ± 61.2	0.098
Word fluency (vegetable)		15.3 ± 12.7	13.5 ± 4	0.436
Word fluency (animal)		16.2 ± 4.7	16.9 ± 4.6	0.228
Word fluency (letter)		6.5 ± 1.7	6.7 ± 1.9	0.17

Cardiac function	Normal range	Baseline	3–6 M	
EF (%)	50–80	64.5 ± 7.2	67.2 ± 7.7	0.025*
LAVI (ml/m^2^)	< 34	46.8 ± 16.9	40.9 ± 13.5	0.002*
BNP (pg/ml)	0–8.4	89.1 ± 70.9	62.5 ± 70.1	0.001*

*DWI* Diffusion-weighted imaging, *DIR* Double inversion recovery, *MCAS* Mie constructional apraxia scale, *EF* Ejection fraction, *LAVI* Left atrial volume index, *BNP* Brain natriuretic peptide.

At baseline, SWI detected 577 MBs. Follow-up SWI after 6 months detected 98 new MBs. Sixty-four out of 98 MBs (65.3%) were exactly at the same location where microinfarctions were found at baseline.

### Correlation between the number of microinfarctions and ablation parameters

The correlation between the number of microinfarctions and duration of the ablation procedure was evaluated, where procedure duration was defined as the time required from the start to end of the ablation. A mild positive correlation was observed (*p* = 0.036, *γ* = 0.248) (Fig. 3D).

### Correlation between MB number and CHADS2 score

We analyzed the correlation between the number of new MBs detected by follow-up MRI and CHADS2 score but observed no significant correlation (*p* = 0.75, *γ* = 0.038).

### Neuropsychological findings after ablation

The MMSE score was 27.9 ± 2.4 at baseline. At 6 months after ablation, the average MMSE score improved significantly to 28.5 ± 1.7 (*p* = 0.037). RBMT (both immediate and delayed recall: *p* < 0.001 and *p* < 0.01, respectively), MCAS (*p* < 0.01), and TMT-A (*p* = 0.001) scores improved significantly at 6 months after ablation (Table 2). To evaluate cognitive enhancement variability, we performed correlation analysis between baseline scores and the change value of MMSE, RBMT immediate recall, RBMT delayed recall, MCAS, TMT-A, which showed significant improvements at 6 months after ablation. Patients with lower baseline cognitive function showed better improvement in almost all these scores (*p* < 0.01, *p* = 0.016, *p* = 0.01, *p* < 0.01, *p* < 0.01, respectively.)

We evaluated the improvement in neuropsychological scores according to history of hypertension, diabetes mellitus, dyslipidemia, and stroke/TIA. There was no significant difference in the changes of any scores between patients with and without hypertension, diabetes mellitus, dyslipidemia, and history of stroke/TIA. Furthermore, we analyzed the difference in cognitive changes between persistent and paroxysmal AF, with and without AF recurrence, with and without oral anticoagulant use at 6 months after ablation, but no difference was observed.

Further, in 20 patients, MMSE was evaluated 1 day before ablation, and the score was 25–30 (average score: 27.9 ± 1.8). We found no significant differences between MMSE scores evaluated before and immediately after catheter ablation (*p* = 0.14).

### Cardiac function after ablation and correlation with neuropsychological findings

**Figure 4:**
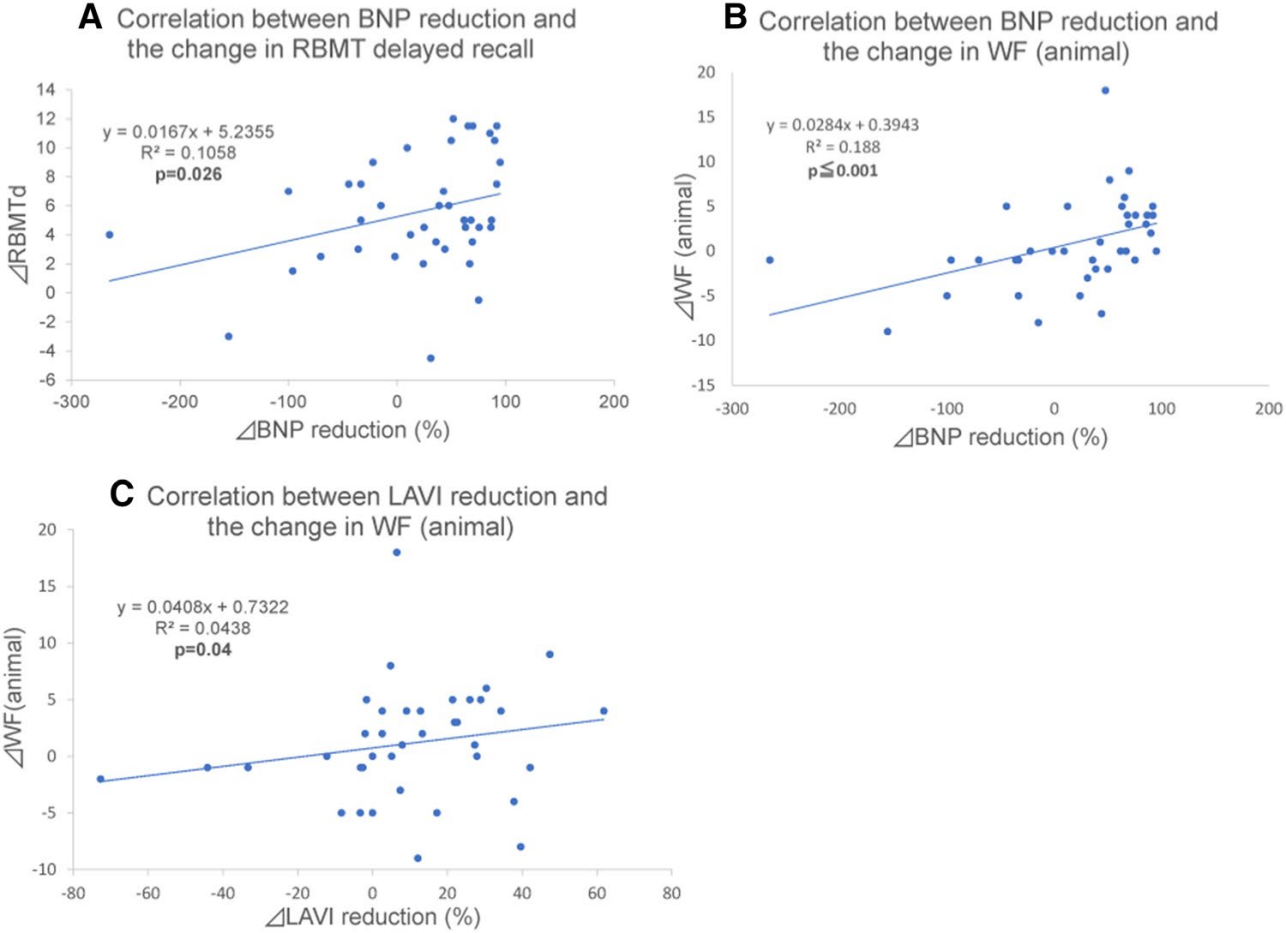
Correlation between cardiac function and neuropsychological findings (n = 40). There were positive correlations between LAVI (%) reduction and word fluency changes (animal) (*p* = 0.04), between BNP (%) reduction and word fluency changes (animal) (*p* ≤ 0.001), and between changes in BNP (%) and those in RBMT delayed recall (*p* = 0.026). We calculated Spearman’s correlation coefficient. *LAVI* Left atrial volume index, *RBMT* Rivermead behavioral memory test, BNP Brain natriuretic peptide, *WF* Word fluency.

EF values significantly increased (*p* = 0.025), whereas LAVI and BNP significantly decreased (*p* = 0.002, *p* = 0.001, respectively) between baseline and at 3–6 months after ablation (Table 2). We compared changes in cardiac function in patients with persistent and paroxysmal AF, with and without AF recurrence within 6 months after ablation, and presence and absence of oral anticoagulant use at 6 months after ablation. No significant difference in cardiac function changes was observed. No correlation was observed between EF percent increase and the changes in MMSE scores or other findings. However, there were positive correlations between LAVI percent reduction and WF changes (animal) (*p* = 0.04, *γ* = 0.331), between BNP reduction and WF changes (animal) (*p* ≦ 0.001,* γ* = 0.546), and between BNP reduction and RBMT delayed recall (*p* = 0.026,* γ* = 0.351) (Fig. 4). In addition, there was no correlation with any neuropsychological score change between the patients who showed LAVI/BNP improvement or not.

## Discussion

Our prospective study indicated that ablation has beneficial effects on overall neuropsychological scores despite the incidental embolic microinfarctions caused by the procedure. After catheter ablation, there was an increase in EF and a decrease in LAVI and BNP, which may be attributable to improved cardiac function, and which might have led to a net beneficial effect on neuropsychological scores.

Both prospective and retrospective studies have examined the relationship between AF and cognitive impairment^27^. AF can become a risk factor of dementia even in the absence of embolic stroke. One of the plausible mechanisms by which AF induces dementia may be chronic cerebral hypoperfusion. It has been suggested that chronic cerebral hypoperfusion is causally related to both AD and VaD^28,29^. AF causes cerebral hypoperfusion through beat-to-beat variability and an overall reduced cardiac output owing to the lack of atrioventricular synchrony^30^. Indeed, cerebral blood flow (CBF) is significantly lower in patients with persistent AF than in those without^31^. Furthermore, the CBF level of patients with paroxysmal AF lies between persistent AF and sinus rhythm^11^, and electrical cardioversion could restore CBF in patients with AF^32^. Cerebral hypoperfusion has been correlated with brain atrophy and dementia^33^, and AF per se is also associated with brain atrophy with a stronger association in persistent/permanent AF than in paroxysmal AF^16^.

The other possible mechanism by which AF induces dementia is by remitting microembolism, which may cause accumulation of cortical microinfarcts (CMIs) and subsequently, dementia^34–36^. Catheter ablation may potentially suppress the chronic incidence of microembolism in AF^37^, while subsequent prevention of microembolism may have a beneficial effect on cognitive dysfunction. However, this possibility could not explain the improvement of neuropsychological scores encompassing overall cognitive domains in the present study, because small vessel diseases, including CMI, usually contribute to frontal lobe dysfunction^38^. Notably, in the present study, improvements were not limited to scores related to frontal lobe function, but universally observed in every cognitive domain including the temporal lobe. This result may indicate that improvement of cardiac output and subsequent CBF might have led to the recovery of neuropsychological scores in patients with AF.

CMIs are caused by various pathological factors such as microembolism, arteriosclerosis, and cerebral amyloid angiopathy^35^. In our study, catheter ablation caused microinfarctions in 85% of patients. This result contradicts earlier published results where microinfarcts were detected in < 30% of patients. The MRI protocol used in this study is very sensitive for detection of microlesions. Indeed, in our MR protocol, DWI slice thickness is 3 mm with no gap. On the other hand, previous studies used a DWI slice thickness of 5 mm with 2 mm gap, so that microinfarcts with 2–3 mm in size could go undetected. Nakamura et al. described that, compared with conventional DWI, thin section (3 mm with no gap) DWI at 1.5 T permitted better lesion conspicuity and more precise stroke diagnosis^39^. Moreover, we used a 3 T MR machine, with better signal-to-noise ratio than 1.5 T. Therefore, we consider that the higher percentage of patients with acute microinfarcts in this study was probably due to the high resolution DWI employed.

Bergui et al. showed that silent embolic microinfarctions after AF ablation were more commonly found in the cerebral cortex^40^. In our study, 80% of microinfarctions were found in the cerebral cortex. After 6 months, a significantly higher proportion of microinfarctions disappeared from the cerebral cortex than from subcortical regions (96.2% vs. 69.1%, respectively) on 3D-DIR images. This result is consistent with the findings of Terge et al., who evaluated the cumulative incidence of acute CMI and found that all acute CMIs disappeared on follow-up MRI (DWI, T1, FLAIR)^41^. Similarly, Havsteen et al. showed that cortical lesions in TIA disappeared more frequently than those in subcortical ones and hypothesized that a strong leptomeningeal collateral circulation in the cortical gray matter may prevent signs of persistent infarction in small gray matter lesions^42^.

Another notable finding is the numerical increase in MBs on follow-up MRIs. Previous studies reported a higher incidence of MBs in patients with AF than in those without^43^, but the pathogenesis of MBs in AF and the relationship between MBs and cognitive function remain unclear. In our study, there was a 17% increase in the total number of MBs during the 6 months after ablation and most de novo MBs corresponded with embolic microinfarctions detected at baseline. Previously, Ito et al. reported 3 cases of de novo lobar MBs transformed from a small cortical infarction^44^. Our results imply that AF-related microemboli may cause CMI, subsequently leading to MBs.

Our present study has several limitations. First, it did not include a control group of AF patients who did not undergo ablation treatment. A memory clinic study showed a significantly higher prevalence of CMIs in the brains of AF patients^45^, and therefore, patients with AF may have had preexisting CMIs. Our study may indicate that most CMIs disappear and that only a small number remains. Second, our study did not perform pre-ablation MRI, so we cannot confirm that all new microinfarctions and MBs detected after ablation were caused by the ablation procedure. However, we conducted pre-ablation MRI in 13 out of 74 patients, none of whom showed new microinfarctions. Moreover, we could not perform pre-ablation neuropsychological assessment in all patients because of time constraints. We conducted a pre-ablation MMSE estimation in 21 patients, among whom we found no significant changes between pre-ablation and post-ablation MMSE scores. We also compared pre-ablation MMSE scores with 6-month follow-up scores in 16 patients (5 out of 21 patients who underwent pre-ablation MMSE dropped out after 6 months), and found that follow-up scores significantly improved (*p* = 0.023). Third, we did not monitor electrocardiography during neuropsychological assessments. Many patients have AF episodes early after ablation, so the influence of arrythmia on neuropsychological scores at 1–3 days after ablation is undeniable. Similarly, the sedation and stress caused by the ablation procedure might also affect cognitive assessment scores, but none of the patients showed any signs of neuropsychological alterations including delirium. As the average hospitalization period after an ablation procedure was 4–5 days, we performed the examinations 1–3 days after ablation and confirmed absence of neuropsychological worsening between pre-ablation period and immediately after ablation in 21 patients. If patients were too ill to perform neuropsychological assessment, we postponed it to the next day or excluded the patients from the study. Fourth, we could not measure cerebral perfusion by CBF single photon emission computed tomography or arterial spin labeling MRI techniques. Further studies are needed to investigate the incidence of embolic microinfarctions in patients with AF and CBF improvements after ablation.

In conclusion, our study showed preserved cognitive function at 6 months after ablation despite the incidence of embolic microinfarctions. The improvement of neuropsychological scores across all cognitive domains might indicate that ablation improves cognitive function by restoring cardiac function and mitigating chronic cerebral hypoperfusion.

## Data Availability

Data are available upon reasonable request from the corresponding author.
